# Nanoscale Reaction Vessels Designed for Synthesis of Copper-Drug Complexes Suitable for Preclinical Development

**DOI:** 10.1371/journal.pone.0153416

**Published:** 2016-04-07

**Authors:** Mohamed Wehbe, Malathi Anantha, Ian Backstrom, Ada Leung, Kent Chen, Armaan Malhotra, Katarina Edwards, Marcel B. Bally

**Affiliations:** 1 Experimental Therapeutics, British Columbia Cancer Research Centre, Vancouver, British Columbia, Canada; 2 Faculty of Pharmaceutical Sciences, University of British Columbia, Vancouver, British Columbia, Canada; 3 Department of Pathology and Laboratory Medicine, University of British Columbia, Vancouver, British Columbia, Canada; 4 Department of Chemistry, University of Uppsala, 3 Husargatan (B7), Uppsala, Sweden; 5 Center for Drug Research and Development, Vancouver, British Columbia, Canada; University of Helsinki, FINLAND

## Abstract

The development of copper-drug complexes (CDCs) is hindered due to their very poor aqueous solubility. Diethyldithiocarbamate (DDC) is the primary metabolite of disulfiram, an approved drug for alcoholism that is being repurposed for cancer. The anticancer activity of DDC is dependent on complexation with copper to form copper bis-diethyldithiocarbamate (Cu(DDC)_2_), a highly insoluble complex that has not been possible to develop for indications requiring parenteral administration. We have resolved this issue by synthesizing Cu(DDC)_2_ inside liposomes. DDC crosses the liposomal lipid bilayer, reacting with the entrapped copper; a reaction that can be observed through a colour change as the solution goes from a light blue to dark brown. This method is successfully applied to other CDCs including the anti-parasitic drug clioquinol, the natural product quercetin and the novel targeted agent CX-5461. Our method provides a simple, transformative solution enabling, for the first time, the development of CDCs as viable candidate anticancer drugs; drugs that would represent a brand new class of therapeutics for cancer patients.

## Introduction

Over the past decade, the number of copper complexes with anticancer activity has been increasing and a number of reviews have detailed their synthesis and development [1–3]. However, copper-drug complexes (CDCs) have not been approved for use in patients. A significant challenge associated with the development of CDCs is their extremely low aqueous solubility, thus making it difficult to establish the utility of these copper-complexes in preclinical models or patients. Further, development of intravenous dosage formulations requires the use of solubilising agents to create products suitable for use. For example, the therapeutic promise of CDCs has largely been based on data obtained with compounds solubilized in dimethyl sulfoxide (DMSO) [4–7]. Here we describe, for the first time, a novel method to prepare formulations of CDCs; formulations that are suitable for intravenous administration.

Our laboratory has previously disclosed methods that rely on copper complexation to encapsulate water-soluble drugs including anthracyclines and camptothecins; however, optimal drug loading and drug retention in these formulations relied on use of both the encapsulated metal and a transmembrane pH gradient [8–11]. In fact, it is established that drugs with protonizable amine functions, can readily be encapsulated in liposomes using a transmembrane pH gradients [12]. Here, liposomes prepared with encapsulated copper were mixed with compounds that have copper-binding moieties. The compounds selected exhibit common attributes of extremely low (< 1 mg/mL) water solubility prior to or after complexation with copper.

**Fig 1:**
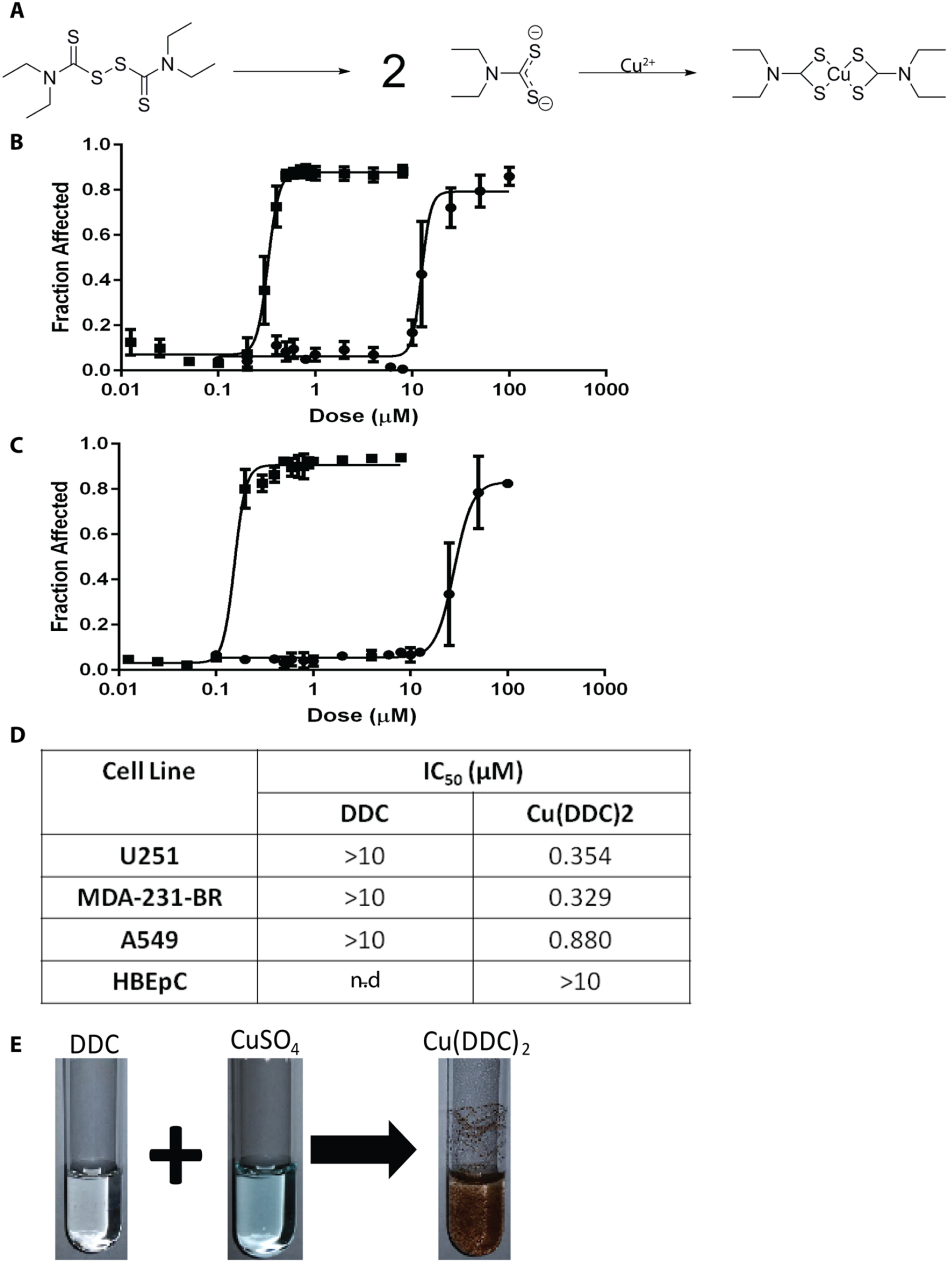
The anticancer activity of diethyldithiocarbmamate (DDC) is dependent on copper. **(A)** Disulfiram is metabolized to diethyldithiocarbamate (DDC) and DDC complexes with Copper (Cu) (II). **(B)** Cytotoxicity curves for DSF (●) and DSF + CuSO_4_ (■) were obtained with the IN CELL Analyzer using U87 glioblastoma cells where cell viability was assessed based on loss of plasma membrane integrity 72 hours following treatment; i.e. total cell count and dead cell count were determined using Hoechst 33342 and ethidium homodimer staining, respectively. **(C)** Cytotoxicity curves for DDC (●) and DDC + CuSO_4_ (■); where cytotoxicity was measured as described above. **(D)** DDC and Cu(DDC)_2_ IC_50_ for U251, MDA-231-BR, and A549 cancer cell lines as well as HBEcP a normal cell line; averages (±SEM) are reported from three separate experiments each done in triplicate. **(E)** Photograph of DDC, CuSO_4_ and Cu(DDC)_2_ solutions in water.

The method was characterized using diethyldithiocarbamate (DDC) (Fig 1) as a model compound; which is known to be the active metabolite generated following administration of disulfiram (DSF) [13]. It has become a drug of interest for use in the treatment of human immunodeficiency virus (HIV) and cancer [14–18]. DSF is metabolized to DDC, a well-known copper chelator [19–21]. DDC forms a copper complex at a 2:1 mole ratio (DDC:Cu^2+^), a reaction that is detected by eye as a brown precipitate forms (Fig 1A). Unlike DDC, Cu(DDC)_2_ is highly insoluble in water. We have been pursuing opportunities related to repurposing DSF for oncology indications and have argued that this effort should focus on Cu(DDC)_2_. Our formulation approach relied on the fact that DDC would be membrane permeable and upon addition to preformed liposomes with entrapped copper salts, complexation would occur within the interior of the liposome. The Cu(DDC)_2_ complex would not be membrane permeable and hence trapped. The resulting formulation was characterized and defined as suitable for intravenous administration.

Importantly, the method for preparing Cu(DDC)_2_ in liposomes proved suitable for other copper complexing compounds. Three types of copper-binding moieties were evaluated including S-Donor, O-Donor and N,O-Donor systems. Examples of drugs that are described here, in addition to DDC (an S-Donor), include Quercetin (Qu) (an O-Donor), Clioquinol (CQ) (an N, O donor) as well as a compound, CX-5461, previously not identified as a copper complexing agent. CQ is an analogue of 8-hydroxyquinole and an approved antibacterial agent. It forms a Cu(II) complex which inhibits proteosome function and is known as a copper ionophore [22, 23]. Qu is a natural product belonging to the class of compounds known as flavanoids. In recent work it was shown to exhibit anticancer effects through generation of reactive oxygen species [24]. CX-5461 is a RNA polymerase inhibitor being evaluated in clinical trials [25, 26] and its use exemplifies the versatility of this method as CX-5461 is a high molecular weight compound with many functional groups capable of binding copper. With the existence of other donor systems not described here, it is likely that this method can be applied to a broad range of drugs and drug candidates with a variety of structures, sizes and functional copper-binding moieties [1, 2].

## Materials and Methods

### Ethics statement

Studies involving the use of animals were completed under an Animal Care Protocol approved by the University of British Columbia's Animal Care Committee. Animals were monitored for changes in body weight, appearance and behaviour. Health assessment was completed using a standard operating procedure (SOP), approved by the Institutional Animal Care Committee. Termination was a result of isoflurane followed by CO_2_ asphyxiation.

### Materials

1,2-distearoyl-sn-glycero-3-phosphocholine (DSPC), Cholesterol (Chol) and 1,2-distearoyl-sn-glycero-3-phosphoethanolamine-n-[carboxy(polyethylene glycol)-2000] (DSPE-PEG_2000_) were purchased from Avanti Polar Lipids (Alabaster, AL) and ^3^H-cholesteryl hexadecyl ether (3H-CHE) from PerkinElmer Life Sciences (Boston, MA). Pico-Fluor 40 scintillation cocktail was purchased from PerkinElmer Life Sciences (Woodbridge, ON, Canada). Disulfiram, Sodium Diethyldithiocarbamate trihydrate, Copper Sulfate, HEPES, Sephadex G-50, Clioquinol, Quercetin (Reagent grade) and all other chemicals were purchased from Sigma Aldrich. CX-5461 was purchased from Selleck Chemicals.

### Cytotoxicity Experiments

The cell lines U87, and A549 were obtained from ATCC, HBEpC (Human Bronchial Epithelial Cells) was obtained from Cell Applications (San Deigo, California) and MDA-231-BR was kindly donated by Patricia Steeg, NIH/NCI. The U251MG glioblastoma cell line (formerly known as U-373 MG) was originally obtained from ATCC (Manassas, VA) and was used for a maximum of fifteen passages. U87, U251MG, A549 and MDA231-BR cells were maintained in DMEM (Gibco) supplemented with 2 mM L-glutamine (Gibco) and 10% fetal bovine serum (Gibco). HBEpC were grown in bronchial/tracheal epithelial growth medium obtained from Cell Applications and were used for a maximum of three passages. All cells were maintained at 37°C and 5% CO_2_. The cells were seeded into 384 well plates and allowed to grow for 24hrs and then treated as specified. After a 72 hr exposure to the indicated compounds the cells were stained with Hoescht 33342 and ethidium homodimer I for total and dead cell counts, respectively. Cells were imaged using an In Cell Analyzer 2200 and cell viability was measured based on viable nuclei count. For these studies DDC was dissolved in sterile water, DSF and Cu(DDC)_2_ were solubilised in DMSO and cells were dosed such that the final DMSO concentration was 0.05%.

### Liposome Preparation

Lipid vesicles (liposomes) were prepared by extrusion [27] and were composed of DSPC/Chol (55:45 mol ratio) or DSPC/Chol/DSPE-PEG_2000_ (45 to 50:45:0 to 5 mole ratio). Briefly, lipids were desiccated for 2 hours after removal from the freezer (-80°C), weighed and dissolved in chloroform at the ratios indicated. The non-exchangeable and non-metabolizable lipid marker ^3^H-CHE was incorporated into the choloroform mixture at a concentration of 12.5–25 nanocurie per μmol lipid. The chloroform was removed under a stream of nitrogen gas prior to being placed under high vacuum for at least 3 hrs to remove residual solvent. The resultant lipid film was hydrated by adding 1 mL of unbuffered 300 mM CuSO4 (pH 3.5) to 50 μmoles of total lipid (final lipid concentration 50 mM) and this was incubated at 65°C with frequent vortex mixing. Subsequently, the hydrated lipids underwent 5 freeze (in liquid nitrogen) and thaw (65°C water bath) cycles. The hydrated lipids were then placed in an Extruder^™^ (Northern Lipids Inc.) and extruded through stacked 0.08 μm polycarbonate filters (Whatman^®^ Nucleopore) 20 times. The size of the resulting liposomes was determined using quasi-electric light scattering (NanoBrook ZetaPALS Potential Analyzer). Prior to adding the specified copper-binding compound, unencapsulated CuSO_4_ was removed by running the sample through a Sephadex G-50 column equilibrated with sucrose (300 mmol/L), HEPES (20 mmol/L) and EDTA (15 mmol) at pH 7.5 (SHE buffer). Subsequently, EDTA was removed by running the sample through a Sephadex G-50 column equilibrated with sucrose (300 mmol/L) and HEPES (20 mmol/L) (pH 7.5) (SH buffer).

### Copper complexation reactions

Copper loaded-liposomes were mixed with DDC (4 or 25°C), CQ (40°C), Qu (50°C) or CX-5461 (60°C) at the indicated compound to liposomal lipid ratio in the SH buffer (pH 7.5) and incubated over a 60-min time course. Liposome and associated compound were separated from unassociated (free) compound using a Sephadex G-50 column equilibrated with SH buffer. The eluted liposome fractions (collected with the excluded volume of the column) were analyzed for copper, compound (as the copper complex or after dissociation of the bound copper) and liposomal lipid concentrations. Lipid concentrations were measured by assaying for [3H]-CHE by liquid scintillation counting (Packard 1900TR Liquid Scintillation Analyzer) where 20 μL of eluted liposome sample was mixed into 5 mLs Pico-Fluor Plus (Perkin Elmer). For the spectrophotometric assay samples were diluted into 1 mL methanol for Cu(DDC)_2_ and Cu(CQ)_2_ and absorbance was measured at 435 nm (1–10 μg/mL) or 275 nm (0.25–2.5 μg/mL), respectively. CuQu and CuCX-5461 were dissolved in 1 mL of 3% acetic acid in methanol and Qu and CX-5461 were measured by assessing absorbance at 372 nm (1–10 μg/mL) or 288 nm (1–10 μg/mL), respectively. Copper was measured using atomic absorption spectrophotomer (AAnalyst600, Perkin Elmer). The Cu-containing liposomes were diluted in 10 mLs of 0.1% HNO_3_. A copper (Cu^2+^) standard curve was generated using Cu^2+^ (0–100 ng/mL) in 2% nitric acid (Sigma Aldrich).

### Characterization of liposomes

All formulations were characterized for size and polydispersity (ZetaPals, Brookhaven). Samples were diluted to 1–5 mM in filtered 0.9% NaCl or SH buffer for size and polydispersity analysis. Further analysis of the Cu(DDC)_2_ formulations was done by cyro-electron microscopy (CEM). CEM analysis was performed using a Zeiss Libra 120 transmission electron microscope at the University of Uppsala, Sweden. Briefly, liposomes were prepared as described above containing either CuSO_4_ or Cu(DDC)_2_ with SH buffer at pH 7.4. In a controlled chamber for humidity and temperature (25°C) samples of 1–2 μL of the sample were deposited on copper grids coated with a holey cellulose acetate butyrate polymer. Excess liquid was blotted away carefully with filter paper and then samples were quickly vitrified by plunging into liquid ethane. This was then transferred to liquid nitrogen to maintain the temperature below 108 K, which minimizes formation of ice crystals. Images were taken in a zero-loss bright-field mode and an accelerating voltage = 80 kV.

### Intravenous administration of CDC formulations

Female CD-1 mice were given bolus tail vein i.v. injections of Cu(DDC)_2_ (15 mg/kg, drug-to-lipid ratio 0.2 mol:mol), Cu(CQ)_2_ (30 mg/kg, drug-to-lipid ratio 0.2 mol:mol), CuQu (70 mg/kg, drug-to-lipid ratio 0.2 mol:mol), or CuCX-5461 (50 mg/kg, drug-to-lipid ratio 0.2 mol:mol). All formulations were prepared using DSPC:Chol (55:45 mole ratio) liposomes with encapsulated 300 mM copper sulfate as described above. To define tolerability of the CDC formulations, mice (n = 3) were given an intravenous injection (lateral tail vein) of the indicated formulation at the specified dose. The health of the animals was measured over a 14 day period after administration and a full necropsy was performed at that time to assess changes in tissue/organ appearance. Pharmacokinetic studies were completed where blood was collected by cardiac puncture in mice terminated at 1, 4, 8 and 24 hours (n = 4 per time point) by isoflurane followed by CO_2_ asphyxiation. Blood was placed into EDTA coated tubes and stored at 4°C until centrifuged at 2500 rpm for 15 min at 4°C in a Beckman Coulter Allegra X-15R centrifuge. Plasma was collected and stored at -80°C until assayed by AAS (see above) for copper, liposomal lipid (see above) or compound as described below.

### Quantification of Cu(DDC)_2_, Clioquinol, Quercetin, and CX-5461

The HPLC assay developed for Cu(DDC)_2_ was not sensitive enough for the *in vivo* studies completed therefore Cu was used as a surrogate marker for Cu(DDC)_2_. Samples were diluted in 0.1% HNO_3_ and subsequently the Cu concentration was measured using AAS (AAnalyst600, Perkin Elmer) as described above. Plasma Cu levels in animals treated with formulated CDCs was corrected for background Cu levels determined in plasma collected from untreated CD-1 mice. All other compounds were measured using HPLC as summarized below using a Waters Alliance HPLC Module 2695 and photodiode array detector model 996 and Empower 2 Software. Clioquinol was measured at 254 nm following separation on a X-terra C18 column (3.5 μm, 3.0 x 150 mm) using a 1:1 mobile phase of water (pH 3 phosphoric acid) and acetonitrile. A 30 μL sample volume was injected, the flow rate was 1 mL/min and column temperature was set at 55°C. Pyrrolidine diethyldithiocarbamate was added to samples and standards at an excess of 3 mol equivalents prior to injection to ensure dissociation of CQ from Cu. Quercetin was measured at 368 nm following separation on a symmetry C18 column (3.5 μm, 3.0 x 150 mm) using a mobile phase of 0.1% TFA in water and acetonitrile (2.3:1). A 25 μL sample volume was injected, the flow rate was set at 1 mL/min and the column temperature was 30°C. Samples and standards were prepared in acidified methanol so as to dissociate the CuQu complex prior to HPLC analysis. Similarly, the quantification of CX-5461 was performed in acidified methanol to dissociate the complex and CX-5461 was measured at 300 nm following separation on a Luna C18 column (5 μm, 4.6 x 150 mm). The mobile phase contained a 1:1.2 mixture of 0.1% TFA in water and 0.1% TFA in methanol. A 5 μL sample volume was injected, the flow rate was set at 1 mL/min and the column temperature was 35°C.

## Results

### Disulfiram, DDC and Cu(DDC)_2_ Cytotoxicity

Disulfiram (DSF) is metabolized to diethyldithiocarbmamate (DDC) (Fig 1A) and DDC is a well-established copper chelator [28, 29]. The cytotoxic activity of DSF when added to cancer cells is copper dependent. As shown in Fig 1B, the IC_50_ of DSF against U87 glioblastoma cells is >10 μM in the absence of copper. In the presence of copper there is a substantial shift (2-orders of magnitude) in cytotoxicity when copper is added with DSF at a 1:1 molar ratio. DSF is unable to interact with copper, thus activity of DSF depends on its degradation to DDC. As shown in Fig 1C, the activity of DDC in the absence of copper is also >10 μM and in the presence of copper (2:1 molar ratio of DDC to copper) is approximately 220 nM. Similar results were obtained in 3 other cell lines where the IC_50_ of copper + DDC was 345, 329 and 880 nM when used against U251 (glioblastoma line), MDA-231BR (a triple negative breast cancer line selected for its propensity to metastasize to the brain) and A549 (lung cancer line) cells, respectively. DDC as well as Cu(DDC)_2_ exhibited little activity when added to normal human bronchial epithelial cells (HBEpC), suggesting specificity of Cu(DDC)_2_ against cancer cells. It is argued that attempts to repurpose DSF as an anticancer drug should focus on Cu(DDC)_2_; however, Cu(DDC)_2_ is almost completely insoluble in aqueous solution (Fig 1E).

### Formation of Cu(DDC)_2_ by addition of DDC to pre-formed copper containing liposomes

**Fig 2:**
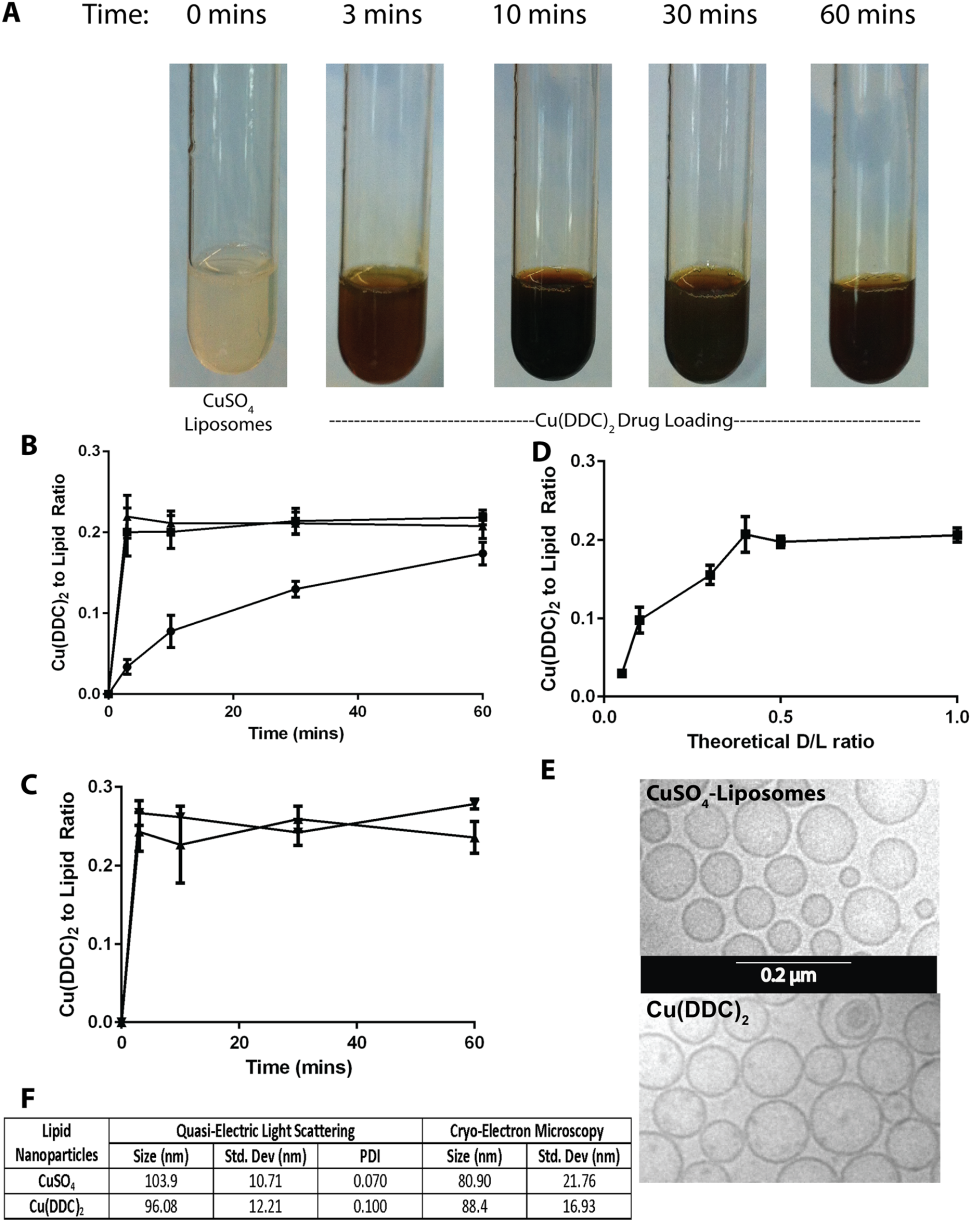
Diethyldithiocarbamate (DDC) loading into DSPC/Chol (55:45) liposomes prepared with encapsulated 300 mM CuSO_4_. **(A)** Photograph of solutions consisting of DDC (5mg/mL) and added to CuSO_4_-containing DSPC/Chol (55:45) liposomes (20 mM liposomal lipid) over a 1 hour at 25°C. **(B)** Formation of Cu(DDC)_2_ inside DSPC/Chol liposomes (20 mM) as a function of time over 1 hour at 4(●), 25(■) and 40(▲)°C following addition of DDC at a final DDC concentration of (5 mM); Cu(DDC)_2_ was measured using a UV-Vis spectrophotometer and lipid was measured using scintillation counting. **(C)** Cu(DDC)_2_ formation inside DSPC/Chol (55:45) liposomes over time where the external pH was 7.4 (▲) and 3.5 (▼). **(D)** Measured Cu(DDC)_2_ as a function of increasing DDC added, represented as the theoretical Cu(DDC)_2_ to total liposomal lipid ratio; where the lipid concentration was fixed at 20 mM and final DDC concentration was varied. **(E)** Cryo-electron microscopy photomicrograph of CuSO_4_- containing DSPC/Chol (55:45) liposomes and the same liposomes after formation of encapsulated Cu(DDC)_2_. **(F)** Size of the CuSO_4_- containing liposomes and liposomes with encapsulated Cu(DDC)_2_ as determined by quasi-electric light scattering and cryo-electron microscopy; data points are given as mean ± SD.

As illustrated in Fig 2, within minutes of DDC addition to copper containing lipid vesicles a color change indicative of Cu(DDC)_2_ formation is visible (Fig 2A). The rate of Cu(DDC)_2_ formation inside the liposome can be quantified by separating liposome-associated Cu(DDC)_2_ from unassociated DDC and then assaying for Cu(DDC)_2_ spectrophotometrically. As shown in Fig 2B, the amount of liposome-associated Cu(DDC)_2_, measured as the Cu(DDC)_2_ to liposomal lipid ratio, is temperature-dependent. Cu(DDC)_2_ association is rapid when DDC is added to copper-containing liposomes at 20°C (RT) and 40°C; where the maximum Cu(DDC)_2_ to lipid ratio of 0.2 (mol ratio) is achieved within 3 minutes. If the temperature is decreased to 4°C, the Cu(DDC)_2_ to lipid ratio of 0.2 (mol ratio) is achieved at 60 minutes. The movement of DDC from the external media into the copper-containing liposomal core is not affected by external pH. As shown in Fig 2C, when the external pH is adjusted to 3.5 the loading rate is comparable to that observed at pH 7.4. To determine the maximum Cu(DDC)_2_ to liposomal lipid ratio that can be achieved when using liposomes prepared in 300 mM copper sulfate, the amount of external DDC was titrated from 0.04 to 0.40 (moles DDC to moles liposomal lipid) and the results suggest that the maximum Cu(DDC)_2_ to lipid ratio achievable under these condition was 0.2 (mol:mol). This was achieved when the initial DDC to liposomal lipid ratio was 0.4 (mol:mol). As indicated, Cu(DDC)_2_ forms an insoluble precipitate and it was possible that the synthesis of Cu(DDC)_2_ inside the liposomes may have engendered formation of a precipitate within the liposomes. To evaluate this, the liposomes were visualized by CEM (Fig 2E). The results illustrate two points: (1) the Cu(DDC)_2_ liposomes exhibited a mean particle size that was comparable to that observed with the copper containing liposomes before addition of DDC, and (2) the formation of Cu(DDC)_2_ inside the liposomes did not result in the formation of an electron dense core. These points would suggest that there is a homogenous distribution of CDCs across all liposomes in solution. It should be noted that the liposome size estimated by CEM analysis was comparable to that determined by QELS (Fig 2F).

**Fig 3:**
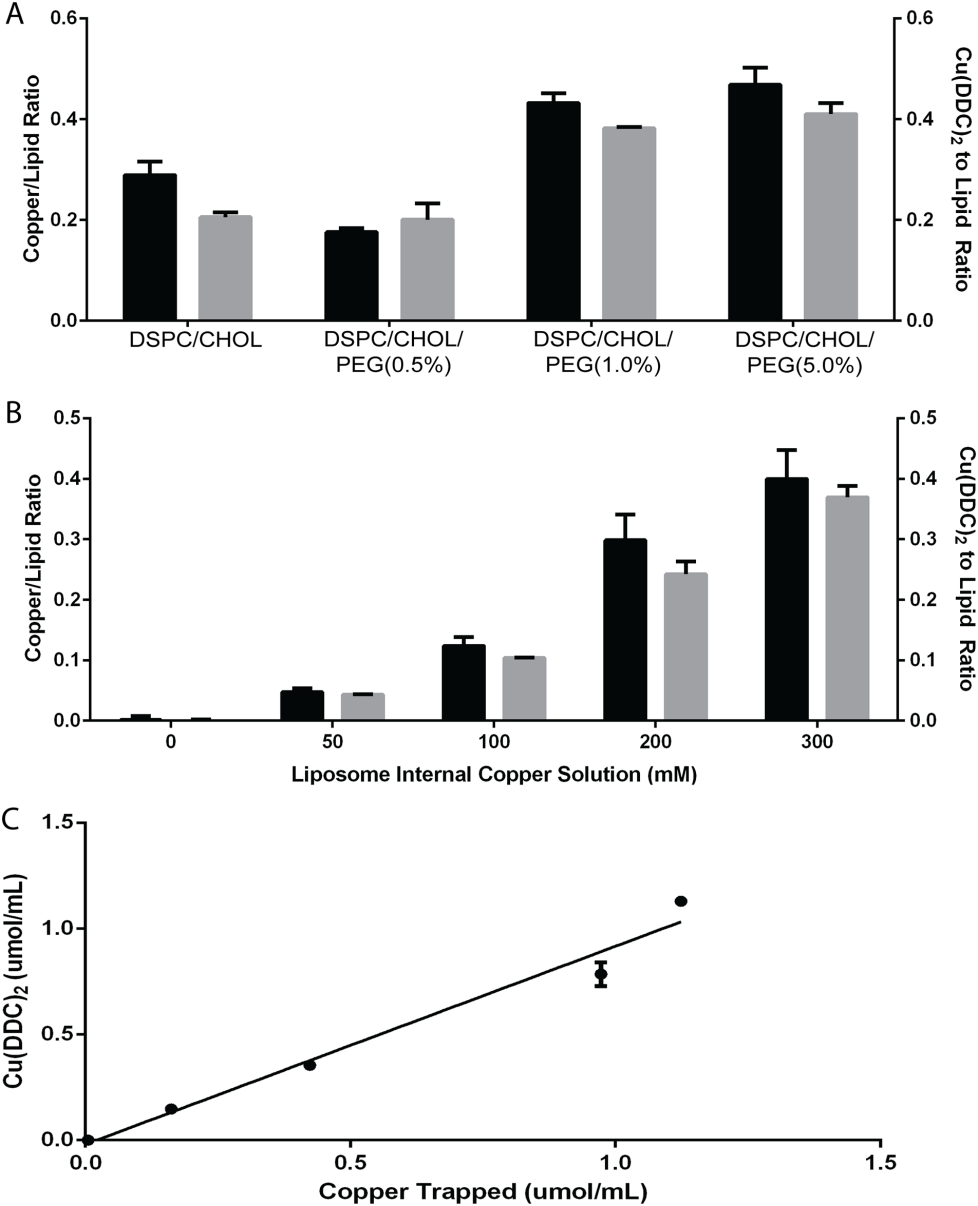
Characterization of copper-complex loading method. **(A)** Measured (AAS) copper to liposomal lipid ratio (black bars) compared to measured Cu(DDC)_2_ (UV-Vis spectrophotometer) to liposomal lipid ratio (grey bars) after DDC was added to CuSO_4_-containing DSPC/Chol liposomes prepared with different amounts of DSPE-PEG_2000_ (ranging from 0 to 5 mole%). **(B)** Formation of Cu(DDC)_2_ inside CuSO_4_-containing DSPC/Chol liposomes as a function of the CuSO_4_ concentration used to prepare the liposomes (ranging from 0 to 300 mM); where the measured copper (AAS) to liposomal lipid ratio (black bar) is compared to the measured Cu(DDC)_2_ (UV-Vis spectrophotometer) to liposomal lipid ratio (grey bar). **(C)** Linear regression analysis comparing measured (AAS) copper concentration (assuming encapsulated copper was free in solution) to measured Cu(DDC)_2_ (UV-Vis spectrophotometer) concentration (assuming encapsulated Cu(DDC)_2_ was free in solution); R^2^ = 0.9754; each data point represents a mean ± SEM determined from at least three separate experiments done in duplicate.

The only change in liposomal lipid composition considered in these studies was incorporation of polyethylene glycol (PEG_2000_) modified DSPE. This was considered for two reasons: (1) PEG_2000_-DSPE is a negatively charged lipid and its use should increase the amount of encapsulated copper when preparing the liposomes; and (2) PEG_2000_-DSPE is known to prevent surface-surface associations that can influence liposome-liposome aggregation and liposome-cell interactions which, in turn, can effect elimination rates *in vivo* [30]. When PEG_2000_-DSPE was added to our base lipid formulation of DSPC:CHOL (55:45) ranging from 0.5 to 5% (based on reductions of DSPC content) the maximum amount of liposome-associated Cu(DDC)_2_ as measured by the Cu(DDC)_2_ to liposomal lipid ratio increased from 0.2 to 0.4 (Fig 3A, black bars). When analyzing the amount of copper associated with these liposomes (gray bars) it was clear that the Cu(DDC)_2_ to liposomal lipid ratio was related to the amount of copper encapsulated in the liposomes. The addition of PEG_2000_-DSPE increased copper encapsulation, likely due to the introduction of an anionic change that is known to enhance liposome trapped volume [27]. The DSPC/CHOL/DSPE-PEG_2000_ (50/45/5 mol ratio) was selected to establish the relationship between amount of encapsulated copper and final Cu(DDC)_2_ to liposomal lipid ratio. These liposomes were prepared using copper sulfate solutions with copper concentrations ranging from 0 to 300 mM. The osmolarity (~300 mOs/kg) of these solutions was balanced with MgSO_4_. These liposomes were analyzed for copper content prior to DDC addition and after addition of DDC in excess (>2-fold molar excess to the measured liposome associated copper for liposomes prepared in the 300 mM copper sulfate solution). The results (Fig 3B and 3C) are consistent with the data in Fig 3A; the Cu(DDC)_2_ to liposomal lipid ratio achieved was directly proportional to the amount of copper retained in the liposomes. A plot of encapsulated copper vs encapsulated Cu(DDC)_2_ demonstrated a linear regression fit of R^2^ = 0.9754. This is consistent with a 1:2 mol ratio between copper and added DDC.

### Use of copper containing liposomes to prepare CDCs suitable for intravenous injection

**Fig 4:**
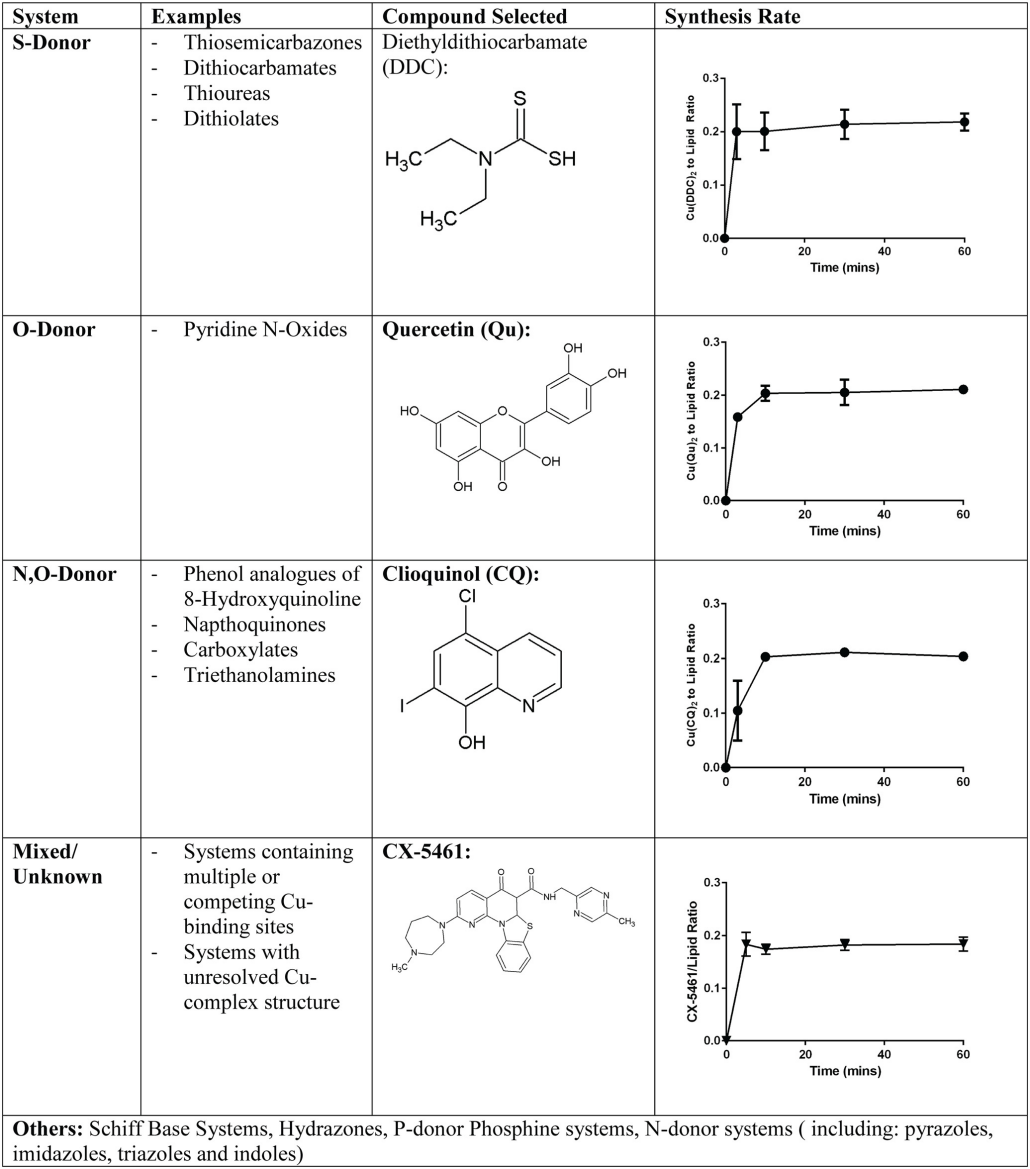
Donor systems that can be used in Copper(II)-complex loading. Copper is able to form complexes with compounds containing S-Donor, O-Donor and N,O-Donor systems as well as other mixed donor systems. Examples of drugs that are described here, in addition to DDC (an S-Donor), include Quercetin (Qu) (an O-Donor), Clioquinol (CQ) (an N,O donor) as well as CX-5461, previously not identified as a copper complexing agent. Each was loaded into DSPC/Chol (55:45 mol ratio) liposomes prepared with 300 mM CuSO_4_. The loading temperature used in these examples was 25, 50, 40 and 60°C, respectively.

The approach is compatible to other copper-binding drugs and candidates. As a platform approach, this method would address one of the most important problems limiting the therapeutic development of copper-drug complexes (CDCs). To assess the breadth of this approach, other compounds that contain functional groups known to bind copper were evaluated. These are summarized in Fig 4 and include, but are not limited to, S-Donor, O-Donor and N,O-Donor systems. Examples tested, in addition to DDC (an S-Donor), include Quercetin (Qu) (an O-Donor), Clioquinol (CQ) (an N,O donor) as well as a compound, CX-5461, previously not identified as a copper complexing agent. The indicated drugs are all sparingly soluble in aqueous solutions at pH 7.4 and all can be encapsulated when added to lipid vesicles (DSPC/CHOL (55:45 mole ratio)) prepared with encapsulated copper. It should be noted that due to their very low aqueous solubility Qu and CQ were added in a solid/powdered form. CX-5461 is also poorly soluble in aqueous solutions but could be prepared as a metastable solution in low pH (4.5) phosphate buffer as described elsewhere [31]. As noted in Fig 4 (far right column) all formulations could be designed to achieve a final Cu-complexed drug to liposomal lipid ratio of 0.2 (mol:mol). In every example formation of the CDC was rapid, but the temperature needed for CDC was empirically determined. Cu(DDC)_2_ formation was optimal at 25°C, Cu(CQ)_2_ formation was optimal at 40°C, the Cu(Qu) and Cu(CX-5461) formations were optimal at 50°C and 60°C, respectively. It should be noted that all the formulations are stable for at least 2 weeks at 4°C (unpublished observation) but more formal stability studies will be required to determine the long term stability.

**Fig 5:**
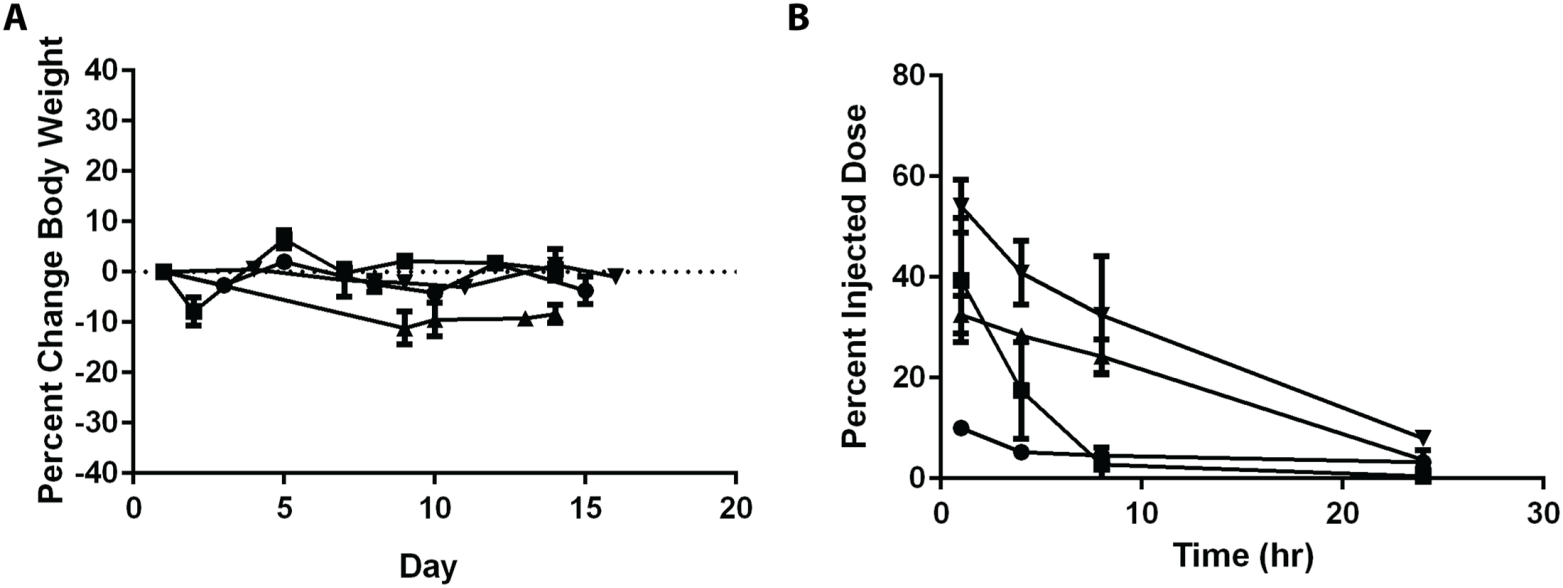
Preliminary tolerability and plasma elimination profiles for liposomal formulations of Cu(DDC)_2_, Cu(CQ)_2_, CuQu and CuCX-5461 after intravenous injection into CD-1 mice. Mice were injected with a single dose of 15 mg/kg Cu(DDC)_2_ (-●-), 30mg/kg Cu(CQ)_2_ (-■-), 70mg/kg CuQu (-▲-) and 50 mg/kg Cu-CX-5461 (-▼-). **(A)** Changes in body weight following administration of the indicated liposomal formulation where body weights were measured over 14 days after injection (n = 3). **(B)** Preliminary plasma elimination profiles of the indicated liposomal formulations where the copper-complexed compound was measured at 1, 4, 8 and 24 hrs after administration (n = 4); concentrations were measured using HPLC or AAS as described in the Methods.

The goal of this method was to facilitate the ability to test copper complex compounds as therapeutics following intravenous administration. Preliminary data to support this is provided in Fig 5. The formulations described in Fig 4, were prepared for single dose safety studies in mice and once a safe dose was defined, the elimination of the copper complex compound was determined as described in the Methods. Fig 5A summarizes the change in body weight of mice injected with the indicated formulation at the determined maximum tolerated dose. The formulations caused <15% body weight loss and other health status indicators suggested mild and reversible changes in animal health status. The elimination behaviors of the intravenously injected compounds were dependent on the compound tested. Cu(DDC)_2_ was eliminated the most rapidly with <10% of the injected dose remaining after 1 hr. Cu(CX-5461) exhibited the longest circulation longevity with > 30% of the injected dose remaining in circulation after 8 hr.

## Discussion

The therapeutic (anti-cancer) activity of DDC (a metabolite generated after administration of DSF) is inactive in the absence of copper; further, DDC is not stable in acidic solutions leading to rapid degradation [32]. The complex formed when mixing CuSO_4_ and DDC is charge neutral and forms as a dark brown precipitate [33]. We proposed that lipid vesicles (liposomes) could behave as “nano-scaled reaction vessels” for Cu(DDC)_2_-complex formation. Provided that the liposome structure remains intact, synthesized Cu(DDC)_2_ would remain in suspension and would be suitable for intravenous administration. This proved to be the case as illustrated by the results in Figs 2 and 5.

The process described here is distinct from other liposomal formulation methods which rely on compounds considered somewhat soluble in aqueous solution (>1mg/mL). Comparisons may be drawn to pH gradient loading methods [10, 34] or comparable methods relying on use of encapsulated ammonium sulfate [35, 36] or encapsulated ions and an added ionophore designed to generate a pH directly [8] or indirectly through formation of a transmembrane potential [37]. In these previous examples, the agents formulated into the liposomes have protonizable amine functions and encapsulation and/or drug retention is influenced by the presence of the transmembrane pH gradient (acidic interior) and/or the presence of residual liposome associated metal. The method described here works for compounds that do not have protonizable amine functions and are sparingly soluble in water. In fact, changing the pH from 7.4 to 3.5 showed no effect on the ability for DDC to complex copper trapped in the lipid vesicles. For the Cu(DDC)_2_ formulation, evidence shows that the amount of Cu(DDC)_2_ formed is directly related to the amount of encapsulated copper (see Fig 3). For DDC, one copper molecule binds two DDC molecules to form Cu(DDC)_2_. This appears to be the case of CQ as well. Whereas, Qu or CX-5461 complexation appears to be defined at a 1 to 1 ratio. Future studies will further characterize what factors influence the nature of the copper complex formed within the liposome.

The rate of copper-complex formation is likely dependent on the rate at which the externally added compound crosses the liposomal lipid bilayer, which in the examples here were prepared from DSPC/Chol. These formulations are generally considered stable and exhibit reduced permeability at physiological temperatures. Formation of Cu(DDC)_2_ is rapid, occurring in minutes, while the reaction between CX-5461 and lipid vesicle entrapped copper is more gradual. The rate of loading may, however, be dependent on several factors, including temperature, divalent metal ion, the counter ion, the ionic strength, pH etc. Using Qu as an example, this compound is added as a powder to the pre-formed copper containing liposomes. The amount of Qu in free solution, albeit low, will increase with increasing temperature. Solubilized Qu will be free to move across the liposomal lipid bilayer (from the outside to the inside), and the permeability of Qu across the membrane will be lipid composition and temperature dependent. Once inside the liposome, Qu will complex with copper, creating a complex that has reduced solubility and reduce membrane permeability.

The preliminary data provided in Fig 5 demonstrates that the rate of release of the CDCs is dependent on the agent used. Each CDC formulation is unique and will be examined more specifically in ensuing publications focusing on *in vitro* and *in vivo* behaviour, including stability of the CDC as well as the retention of the active agents. Cu(DDC)_2_ is released rapidly from the liposomes following i.v. administration of the formulations as evidenced by the rapid elimination of Cu(DDC)_2_. The rate of elimination of Cu-complexed CX-5461 is considerably slower. Although not shown, it is important to note that both copper and complexed agent are released when administering the Cu(DDC)_2_ and Cu(CQ)_2_ formulations. In contrast, it appears that only Qu and CX-5461 are released from the Cu(Qu) and Cu(CX-5461) formulations in a form that is not complexed to copper. This has only been established in lipid vesicles prepared from DSPC/Chol (55:45, mole ratio) and further study into the effects of lipid composition and copper salt will be performed.

By formulating complexes through an inorganic synthesis reaction occurring within the liposomal core, we can obtain high drug-to-lipid ratios that are dependent on the number of copper ions inside and the nature of the complex formed. The formulations prepared this way appear stable; all formulations described here were stable with respect to particle size, polydispersity and complex to liposomal lipid ratio for at least 21 days at 4°C (results not shown). The method is scalable and suitable for manufacturing a pharmaceutical product. The inability to test poorly soluble complexes is a problem that has limited the ability of promising copper-based therapeutics to transition from the lab to the clinic. There are studies that show efficacy in tumour models using solubilising agents that have been formulated with other strategies like very low pH or Cremphor/DMSO/Ethanol mixtures [38–40]; solutions that are not particularly suitable for human use.

## Conclusion

Our method provides a simple, transformative solution enabling, for the first time, the development of CDCs as viable candidate anticancer drugs; drugs that would represent a brand new class of therapeutics for cancer patients. The focus of this method development was on the synthesis of Cu(DDC)_2_ in lipid vesicles and the resulting product is the first injectable Cu(DDC)_2_ formulation described in the literature. The process has been extended to other compounds including clioquinol, quercetin and CX-5461. In each case the candidate CDC formulation appears stable and address problems related to the solubility for each agent. This allowed for the preclinical assessment of these CDCs and it will be expanded to other agents.
